# Case Report: IgG4-rich tubulointerstitial inflammation in MPO-ANCA-associated glomerulonephritis: a case-based review

**DOI:** 10.3389/fimmu.2026.1708062

**Published:** 2026-05-12

**Authors:** Songlin Qiu, Sufen Li, Minya Jin, Yijun Chen, Suyun Chen

**Affiliations:** 1 Department of Clinical Laboratory, Taizhou Hospital of Zhejiang Province Affiliated to Wenzhou Medical University, Linhai, China; 2 Key Laboratory of System Medicine and Precision Diagnosis and Treatment of, Taizhou, China; 3 Department of Pathology, Taizhou Hospital of Zhejiang Province Affiliated to Wenzhou Medical University, Linhai, China

**Keywords:** ANCA-associated glomerulonephritis, clinicopathological phenotype, diagnostic pitfalls, IgG4-related tubulointerstitial nephritis, pauci-immune crescentic glomerulonephritis

## Abstract

Immunoglobulin G4-related disease (IgG4-RD) is a systemic, immune-mediated fibro-inflammatory disorder with the potential to affect multiple organs, with the kidney being the most commonly involved organ, typically presenting as IgG4-related tubulointerstitial nephritis (IgG4-TIN). Anti-neutrophil cytoplasmic antibody (ANCA)-associated glomerulonephritis (ANCA-GN), in contrast, is a pauci-immune necrotizing crescentic glomerulonephritis characterized by acute renal dysfunction and systemic inflammation. While early reports have interpreted IgG4-positive plasma cell infiltration in ANCA-associated vasculitis as evidence of overlap with IgG4-TIN, histologic resemblance in varying disease contexts can make differentiation challenging. In this context, we present the case of an 81-year-old woman diagnosed with both ANCA-GN and IgG4-TIN, informed by a comprehensive review of all previously reported cases. Renal biopsy confirmed this rare overlap, revealing pauci-immune crescentic glomerulonephritis alongside dense IgG4-positive plasma cell infiltration and storiform fibrosis. This case illustrates the diagnostic complexity posed by IgG4-rich inflammatory infiltrates in ANCA-associated disease and underscores the importance of careful clinicopathologic correlation to guide accurate diagnosis, appropriate immunosuppressive therapy, and improved understanding of potentially overlapping autoimmune mechanisms.

## Highlights

- We report on an elderly patient with myeloperoxidase–anti-neutrophil cytoplasmic antibody (MPO-ANCA)-associated glomerulonephritis demonstrating prominent immunoglobulin G4 (IgG4)-positive plasma cell infiltration and storiform fibrosis.
- Renal biopsy revealed both pauci-immune crescentic glomerulonephritis and typical IgG4-related features, including storiform fibrosis and IgG4-positive plasma cell infiltration.
- This case highlights the diagnostic challenge posed by IgG4-rich inflammatory infiltrates in ANCA-associated vasculitis and underscores the importance of careful clinicopathologic correlation.

## Introduction

Immunoglobulin G4-related disease (IgG4-RD) is a systemic fibroinflammatory disorder that can involve multiple organ systems. Renal involvement typically presents as IgG4-related tubulointerstitial nephritis (IgG4-TIN), which is histologically characterized by a dense infiltration of IgG4-positive plasma cells and distinctive storiform fibrosis within the renal interstitium (1). Less commonly, glomerular involvement has also been reported, including PLA2R-negative membranous nephropathy, highlighting the heterogeneity of IgG4-related kidney disease (IgG4-RKD) (2). However, the concurrent occurrence of IgG4-TIN with other autoimmune kidney diseases is infrequently documented.

Anti-neutrophil cytoplasmic antibody (ANCA)-associated vasculitis (AAV) represents a significant form of systemic vasculitis with a marked affinity for renal tissue, resulting in rapidly progressive glomerulonephritis (RPGN) in over 75% of affected individuals (3). The resultant ANCA-associated glomerulonephritis (ANCA-GN) is a clinically relevant subtype of pauci-immune crescentic glomerulonephritis characterized by a paucity of immunoglobulin deposits on renal biopsy and the presence of ANCA in 80%–90% of patients (4–6). Given its poor renal prognosis and rising incidence in the elderly population, the clinical significance of ANCA-GN is substantial (7). Recent case reports have documented ANCA-GN with IgG4-positive plasma cell infiltration, which may suggest a potential overlap with IgG4-RD.

This report presents a detailed case of an 81-year-old woman with myeloperoxidase–ANCA-associated glomerulonephritis (MPO-ANCA-GN) accompanied by prominent IgG4-rich tubulointerstitial inflammation. Through comprehensive analysis of her clinical presentation, laboratory findings, and renal biopsy pathology, together with a review of the relevant literature, we examine the diagnostic complexity arising from the concurrence of pauci-immune crescentic glomerulonephritis and IgG4-positive plasma cell-rich interstitial inflammation.

This case highlights the importance of careful clinicopathologic correlation when IgG4-related histologic features are observed in the setting of AAV. Currently, available evidence does not permit definitive determination of whether such findings represent two distinct disease processes or an IgG4-enriched inflammatory phenotype within AAV. Therefore, comprehensive and multidisciplinary evaluation remains essential to ensure accurate diagnosis and appropriate therapeutic decision-making.

## Case presentation

An 81-year-old female patient was admitted on April 14, 2025, with a 6-day history of bilateral lower extremity edema. Her past medical history included type 2 diabetes mellitus, osteoporosis, lumbar disc herniation, and a prior surgical repair of a T12 thoracic vertebral fracture. On admission, her temperature was 36.7°C, blood pressure was 131/67 mmHg, and physical examination revealed significant and symmetrical pitting edema of the lower extremities. Cardiopulmonary and abdominal examinations were otherwise unremarkable. Review of prior laboratory records from our institution over the past several years demonstrated stable renal function, with serum creatinine values ranging from 58 to 84 μmol/L (68, 58, 72, 84, 70, and 62 μmol/L), without a progressive upward trend, indicating no evidence of preexisting chronic kidney disease.

Initial laboratory findings were significant for elevated serum creatinine of 149 μmol/L, blood urea nitrogen of 15.74 mmol/L, and a calculated estimated glomerular filtration rate (eGFR) of approximately 28 ml/min per 1.73 m^2^. Other results included hyperuricemia (471 μmol/L), hypoalbuminemia (29.8 g/L), mild anemia (hemoglobin, 111 g/L), and a markedly elevated high-sensitivity C-reactive protein (CRP; 101.3 mg/L).

Urinalysis showed 3+ proteinuria, 3+ occult blood, and 3+ glucose, with an elevated red blood cell count (60/μl) and white blood cell count (83/μl). Immunological workup was notable for a high anti-MPO antibody level of 142.20 AU/ml (with a negative anti-PR3 antibody), an elevated serum IgG4 level of 3.97 g/L, a decreased complement C3 (0.58 g/L) with normal C4, elevated C1q at 244 mg/L (reference range, 159–230 mg/L), and rheumatoid factor of 93.2 KU/L (reference range, <14.0 KU/L). Screenings for tumor markers, hepatitis B/C, and HIV and a T-SPOT.TB test were all negative.

Imaging studies revealed multiple pulmonary nodules, a left renal calculus, and a small amount of pelvic effusion. The patient also had poorly controlled blood glucose, with a fasting blood glucose of 16.27 mmol/L and an HbA1c of 8.2%.

## Pathology findings

**Figure 1:**
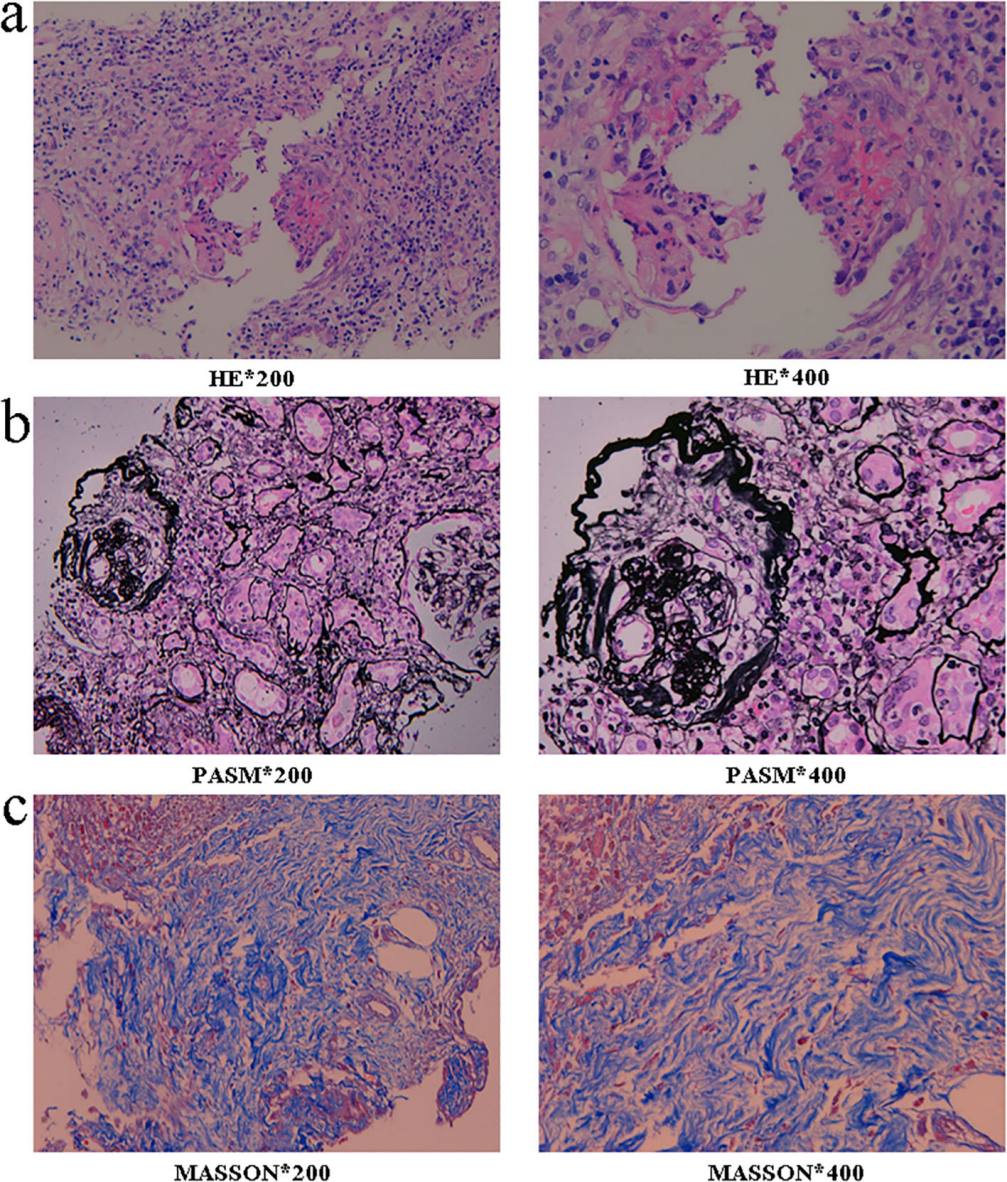
Renal biopsy pathology findings. **(a)** Large cellular crescent formation within the glomerulus, accompanied by infiltration of mononuclear cells in the capillary loops. Note the patchy infiltration of monocytes, lymphocytes, plasma cells, and neutrophils; **(b)** Glomerular sclerosis; **(c)** Patchy interstitial fibrosis.

The biopsy specimen contained one core each of cortical and medullary tissue, in which eight glomeruli were observed. Of these, three showed global sclerosis, while one presented with a large cellular crescent and mononuclear cell infiltration within the capillary loops (**Figures 1A, B**). Two glomeruli displayed parietal epithelial cell proliferation. The remaining glomeruli showed mild proliferation of mesangial cells and matrix. Standard staining with Masson’s trichrome, periodic acid–Schiff (PAS), and periodic acid–methenamine (PASM) did not reveal any evidence of basement membrane thickening.

The renal interstitium showed focal fibrosis, occupying less than 25% of the sample (**Figure 1C**). A diffuse infiltration of mononuclear cells, lymphocytes, plasma cells, and neutrophils was observed, involving over 75% of the interstitium (**Figure 1A**). There was also focal tubular atrophy affecting less than 25% of the tubules, accompanied by granular and vacuolar degeneration of the tubular epithelial cells. Protein casts and red blood cell casts were also present.

### Immunofluorescence analysis

**Figure 2:**
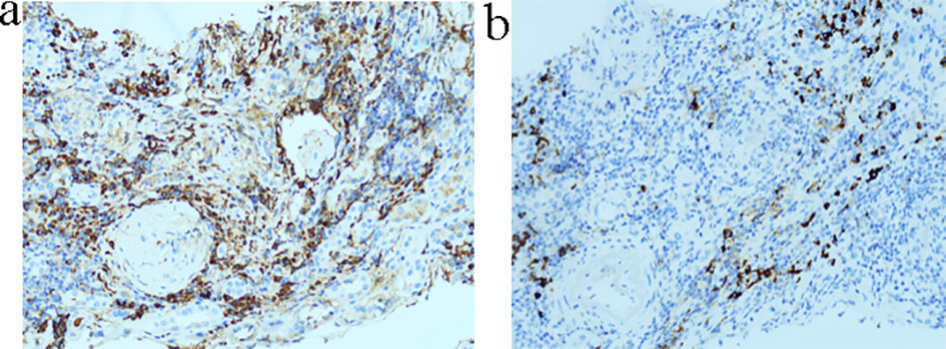
Renal biopsy immunohistochemistry findings. **(a)** Strong positive staining for IgG. **(b)** More than 10 IgG4-positive cells per highpower field (HPF).

Immunofluorescence (IF) analysis was performed on one to three glomeruli and showed negative staining for IgA, IgG, C3, C4, C1q, fibrinogen, IgG1, IgG2, IgG3, IgG4, kappa, lambda, and PLA2R. Only IgM demonstrated weak positive staining in a few glomeruli, which is consistent with a pauci-immune pattern. Special staining, including Congo red with and without oxidation, was negative. Immunohistochemistry (IHC) demonstrated strong positive staining for IgG, with the number of IgG4-positive cells exceeding 10 per high-power field (HPF) (**Figures 2A, B**). The IgG4/IgG ratio was greater than 40%.

**Figure 3:**
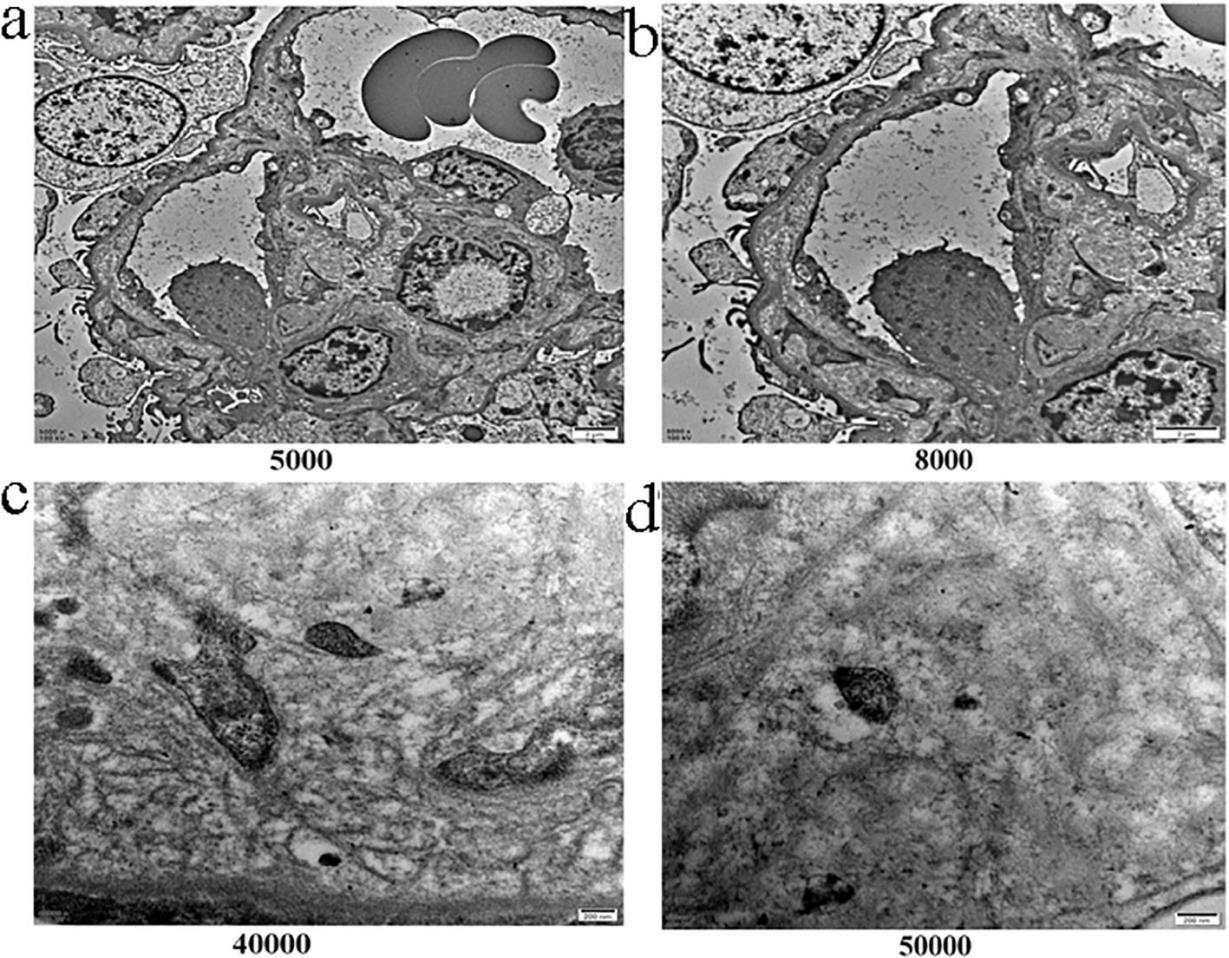
Electron microscopy findings. **(a)** Electron micrograph (°¡5,000) of the glomerulus showing capillary loops. **(b)** Higher magnification view (°¡8,000) showing effacement of podocyte foot processes. The glomerular basement membrane (GBM) appears normal in thickness. **(c)** High-magnification electron micrograph (°¡40,000). **(d)** Ultra-high magnification view (°¡50,000).

Electron microscopy showed well-patent glomerular capillary loops with no significant endothelial cell proliferation (**Figure 3A**). The glomerular capillary basement membrane thickness ranged from 260 to 300 nm, with a maximum thickness of approximately 400 nm. The subendothelial space of the glomerular capillary basement membrane was loose and widened, with flocculent material visible. Mesangial cell and matrix proliferation were present in the glomerular mesangium, and there was approximately 60% effacement of podocyte foot processes (**Figure 3B**). There was no significant proliferation of parietal epithelial cells in Bowman’s capsule, and no crescent formation was observed. Crucially, no definitive electron-dense deposits were found in the glomerular mesangial area, subendothelial or intramembranous spaces of the glomerular basement membrane, or the subepithelial space (**Figures 3C, D**).

### Diagnostic evaluation, treatment, and outcomes

To distinguish IgG4-RKD with incidental MPO-ANCA positivity from true coexisting MPO-ANCA-GN, we integrated histopathological, serological, and clinical findings. Renal biopsy demonstrated crescentic glomerulonephritis in the absence of definite necrotizing lesions; nevertheless, this pattern is more characteristic of AAV than IgG4-RKD alone. In addition, the markedly elevated MPO-ANCA titer (142.20 AU/ml) and the significantly increased high-sensitivity CRP (101.3 mg/L) support active systemic vasculitic inflammation.

Conversely, the presence of >10 IgG4-positive plasma cells per HPF and storiform fibrosis in the renal interstitium are typical features of IgG4-related fibroinflammatory disease. The coexistence of these glomerular and interstitial patterns, together with elevated serum IgG4 levels, suggests a dual immune process rather than incidental MPO-ANCA positivity in isolated IgG4-RKD. Therefore, this case was interpreted as MPO-ANCA-GN with prominent IgG4-related histopathological features, representing a possible overlap rather than simple immunologic mimicry.

**Figure 4:**
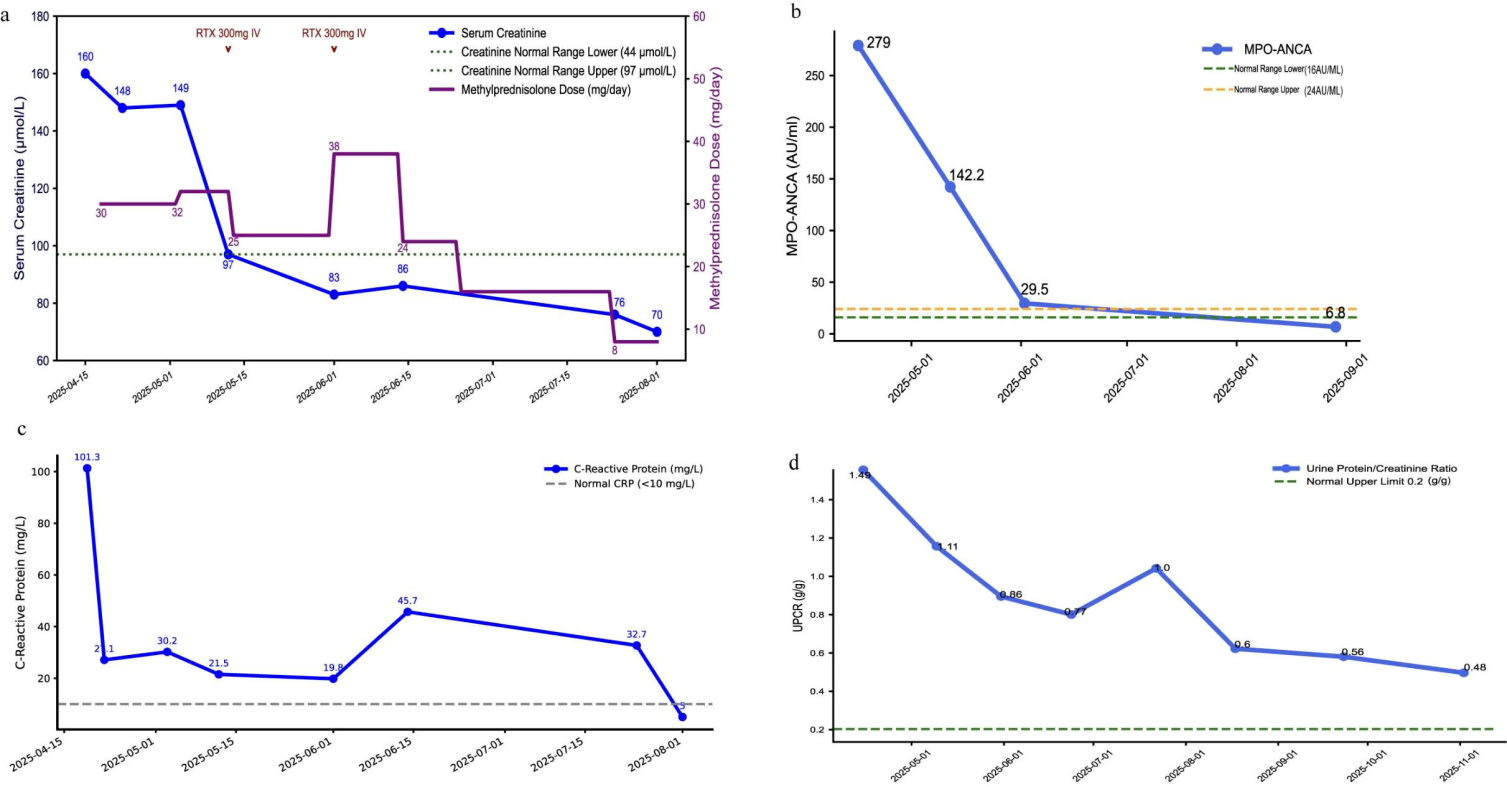
Longitudinal monitoring of the clinical and immunological parameters. **(a)** Serum creatinine levels and therapeutic regimen, including rituximab (RTX) infusions and methylprednisolone dosage. **(b)** Myeloperoxidase–anti-neutrophil cytoplasmic antibody (MPO-ANCA) titers showing seroconversion post-treatment. **(c)** C-reactive protein (CRP) levels indicating resolution of systemic inflammation. **(d)** Trends in the urine protein/creatinine ratio (UPCR). *Dashed lines* in all panels represent respective normal reference ranges.

Upon admission, the patient was started on methylprednisolone (MP; 32 mg/m^2^) combined with a single infusion of rituximab (RTX; 300 mg). She was subsequently maintained on MP (28 mg/m^2^) for ongoing immunosuppression and received supportive care, including insulin for glycemic control (**Figure 4A**). Following treatment, her renal function stabilized, proteinuria was significantly reduced, and bilateral lower extremity edema showed a marked improvement before discharge (**Figure 4B**).

Serial assessments from April 15 to August 1 demonstrated sustained renal recovery and immunological remission (**Figure 1**). Serum creatinine declined from 160 μmol/L at baseline to 70 μmol/L by the final evaluation, with a marked reduction within the first month (**Figure 1A**). Concurrently, the urine protein/creatinine ratio (UPCR) decreased from 1.49 to 0.48 g/g (**Figure 1D**). The MPO-ANCA titers exhibited a rapid and profound reduction from 279 to 6.8 AU/ml, achieving complete seroconversion to below the upper limit of normal (**Figure 1B**). The CRP levels dropped from 101.3 mg/L at presentation to 5 mg/L, ultimately normalizing despite minor transient fluctuations during therapy adjustment (**Figure 1C**). These longitudinal data during treatment illustrate a sustained and favorable therapeutic response.

## Literature review

From a review of the literature, we identified 30 patients with ANCA-GN and renal impairment, some of whom displayed IgG4-positive plasma cell infiltration suggestive of a potential overlap with IgG4-RD. The cohort consisted of 19 men and 11 women, with a mean age of 60.57 ± 11.98 years. All included cases were positive for MPO-ANCA and underwent a renal biopsy, which showed the typical pathological features of pauci-immune necrotizing crescentic glomerulonephritis. Although some of the patients were clinically diagnosed with AAV, their renal biopsy findings were essentially consistent with the pathological characteristics of ANCA-GN, with the kidney as the sole organ involved. Therefore, these cases were uniformly included in our analysis to facilitate a comparison of the commonalities and differences in IgG4-related renal damage.

All patients in the study met the diagnostic criteria for IgG4-RD, demonstrating a prominent pathological feature of an IgG4+/IgG+ plasma cell ratio greater than 40% and an IgG4+ plasma cell count exceeding 10 per HPF in their biopsy tissues. Their serum IgG4 levels were also notably elevated above 1.35 g/L, with a mean value of 8.11 ± 12.51 (7, 8).

All patients were positive for MPO-ANCA. Specifically, 23 patients were diagnosed with ANCA-GN combined with IgG4-TIN, while the remaining seven patients were diagnosed with ANCA-GN due to AAV with renal involvement. The presenting symptoms were primarily acute kidney injury, hematuria, proteinuria, and bilateral lower limb edema, with only two patients presenting with nonspecific symptoms such as fever, fatigue, or upper abdominal pain. Accompanying laboratory findings consistently showed a significant elevation in serum creatinine. In addition, some patients had markedly elevated CRP levels, with a mean of 63.20 ± 44.12.

For the majority of patients, the primary treatment strategy involved a combination of a glucocorticoid (GC) and cyclophosphamide (CYC). A typical regimen began with intravenous (IV) pulse therapy of MP or prednisone (PS), followed by oral GC. This was administered in conjunction with either monthly IV or daily oral CYC. RTX was another therapeutic option, used either in combination with GC or as part of a triple-therapy regimen with GC and CYC in severe cases.

Less common strategies included GC monotherapy and a unique individualized regimen consisting of GC, RTX infusions, and oral *Tripterygium wilfordii* glycosides (TGP). For severely ill patients, plasma exchange (PLEX) was used as an adjunctive treatment. Renal replacement therapy (RRT) was initiated for patients who progressed to end-stage renal disease.

## Discussion

AAV is the most common cause of RPGN worldwide, and a renal biopsy is considered the gold standard for diagnosing ANCA-GN. On histopathology, ANCA-GN typically shows a pauci-immune deposition pattern on IF (9). Here, we report on a case of MPO-ANCA-positive pauci-immune crescentic glomerulonephritis with overlapping IgG4-TIN, where the renal biopsy findings met the diagnostic criteria for both diseases.

To better understand the characteristics of patients with coexisting IgG4-TIN and ANCA-GN, we conducted a systematic review of the existing literature and data from our center. All included patients underwent a renal biopsy, and the histological findings were consistent with the classic pathological changes of both IgG4-RD and ANCA-associated nephritis. These changes included interstitial inflammation rich in IgG4-positive plasma cells and necrotizing crescentic glomerulonephritis. The findings indicate that individuals with this overlap syndrome often present with severe renal dysfunction and marked systemic inflammation, with renal histology showing overlapping features of both IgG4-RD and ANCA-GN.

Previous studies have shown that ANCA-GN is most common in individuals over 60 years of age, with its incidence rising significantly after age 50 (10). In this case series, we observed several shared clinical features in patients with concomitant ANCA-GN and IgG4-RKD. The cohort had a mean age of 60.57 ± 11.98 years and a male predominance, suggesting a specific age and sex predilection for this overlap syndrome. The primary presenting symptoms of IgG4-TIN are typically acute or chronic kidney injury (**Table 1**) (11). Similarly, our patient showed a consistent and severe clinical phenotype at initial presentation, with the most common symptoms being hematuria, proteinuria, and lower limb edema resulting from acute kidney injury. Key laboratory features included acute kidney injury (mean serum creatinine, 312.8 µmol/L), a significant systemic inflammatory response (mean CRP, 63.2 mg/L), and markedly elevated serum IgG4 levels (mean, 8.11 g/L). These findings collectively indicate that these patients frequently present with both severe renal dysfunction and a highly active systemic inflammatory state.

**Table 1:** Characteristics of 30 patients with anti-neutrophil cytoplasmic antibody-associated glomerulonephritis (ANCA-GN) and immunoglobulin G4-related disease (IgG4-RD) overlap.

Author (reference)	No.	Sex	Age (years)	Clinical manifestation	MPO titer (IU/ml)	IgG4-RD	IgG4-positive plasma cell	ANCA-GN or AAV	Renal biopsy	Clinical laboratory metrics			Proteinuria	Microscopic hematuria	Treatment	Outcome
										IgG4 (g/L)	SCr (µmol/L)	CRP (mg/L)				
Wu (27)	1	F	58	Acute kidney injury, hematuria, and proteinuria	55	Confirmed	IgG4 deposits with more than 10 IgG4-positive plasma cells per high-power field	ANCA-GN	Consistent with pauci-immune glomerulonephritis	1.94	227	102	0.8 g/24 h	<50 (RBCs/HPF)	CYC pulse therapy (initial dose of 1 g), followed by subsequent planned CYC pulses combined with continuous oral PS (30 mg/day)	The patient was in good condition, showing significant improvement in both renal function and inflammatory markers. Her serum IgG4 level normalized.
Su (12)	1	M	42	Recurrent epigastric pain	200	confirmed	An IgG4+ plasma cell ratio of over 40%	ANCA-GN	Severe destruction of glomerular capillary loops, cellular crescent formation, and rupture of Bowman’s capsule	1.83	157	79.4	–	10–15 (RBCs/HPF)	MP pulse therapy (0.5 g/day for 3 days) × 2 courses followed by oral PS (1 mg kg−1day−1) with a gradual taper combined with monthly intravenous CYC (0.6 g) and RTX (500 mg)	Renal function showed improvement.
Li (19)	19	12M/7F	62.8 ± 9.4	Acute kidney injury or hematuria	–	Confirmed	Increased infiltration of IgG4+ plasma cells, with a mean count of 68.0 ± 49.0 per high-power field (HPF)	ANCA-GN	Pauci-immune necrotizing or crescentic glomerulonephritis	4.3 ± 2.4	320.9 ± 191.4	–	–	Absent or minimal	GC monotherapy: 2 patients GC + CYC: 16 patients GC + RTX: 1 patient	Six patients had complete remission and nine had partial remission, with one patient relapsing. Four patients did not respond to treatment, and three of them started maintenance hemodialysis.
Wang (28)	3	M	54/56/43	Nausea, loss of appetite, and acute kidney injury	168.0/121.1/114.1	Confirmed	More than 10 IgG4+ plasma cells per high-power field (HPF)	AAV	Necrotizing crescentic glomerulonephritis	7.32/4.26/2.19	413/274.4/905.5	36.95/35.79/36.93	2.38/1.11/0.57 (g/day)	8–15/5–10/full (RBCs/HPF)	MP pulse therapy (0.5 g/day for 3 days), followed by oral PS (initial dose of 40–50 mg/day), with a gradual taper combined with CYC therapy. PLEX was refused by the patient due to financial reasons.	Renal function did not recover, and the patient remains on maintenance dialysis.
He (29)	1	M	63	Emaciation, fatigue, foamy urine, and elevated serum creatinine	>200	Confirmed	More than 10 IgG4+ plasma cells per high-power field (HPF), with an IgG4+/IgG+ plasma cell ratio of over 40%	AAV	Pauci-immune crescentic glomerulonephritis	7.32	413.0	13.8	1.81 g/24 h	–	Induction therapy with IV MP followed by maintenance therapy with oral PS, which was gradually tapered.	During the 3.5-month follow-up period, the patient remained asymptomatic with near-normal renal function. Serum IgG4 levels decreased to 5 g/L.
Capecchi (18)	1	M	74	Chronic cough, hoarseness, and dysphagia	/	Confirmed	An IgG4+/IgG+ plasma cell ratio of >40%, with 45 IgG4+ plasma cells per high-power field (HPF)	AAV	Extracapillary proliferation, leading to the rupture of Bowman’s capsule	45.29	–	21.6	100 mg/dl	Micropyuria, and microhematuria	IV PS therapy (500 mg/day for 3 days), followed by monthly IV CYC at a dose of 1 g per pulse (for a total dose of 7 g).	After 6 months, the patient’s creatinine level was 1.4 mg/dl, with renal function having returned to normal.
Feng (30)	1	M	72	Intermittent fever and fatigue	203.45	Confirmed	IgG4-positive plasma cells comprising more than 40% of the IgG-positive cells	ANCA-GN	67% of glomeruli showed crescentic scarring, accompanied by segmental cytoplasmic necrosis, with no significant immune deposits	7.230	233.9	141	1.24 g/24 h	3+ occult blood	Initiated with oral MP at 120 mg/day, which was subsequently tapered to a maintenance dose. Added on day 10 of GC therapy. An initial dose of 0.4 g was administered, and the regimen was adjusted to 0.6 g monthly after 15 days.	No disease relapse was observed during the subsequent 7-month follow-up period.
Martín-Nares (31)	1	F	69	Fever	–	Uncertain	–	AAV	Pauci-immune complex proliferative extracapillary and necrotizing glomerulonephritis	–	–	–	–	–	GC+RRT	No response
Lu (32)	1	F	54	Bilateral lower limb weakness and myalgia, low-grade fever, cough, and bilateral lower limb edema	89.51	Confirmed	An IgG4+ cell count of 132 per high-power field	AAV	Pauci-immune complex proliferative extracapillary and necrotizing glomerulonephritis	>3.53	375	–	Proteinuria	Microhematuria	MP pulse therapy for 3 days, followed by oral PS (1 mg kg−1 day−1) combined with an IV CYC pulse (400 mg).	The patient refused further treatment and, at a 6-month follow-up, progressed to renal failure and subsequently died.
Present case	1	F	81	Significant bilateral symmetrical pitting edema of the lower extremities	142.20	Confirmed	Increased infiltration of IgG4+ plasma cells (>10/HPF), with an IgG4+/IgG plasma cell ratio of >40%	ANCA-GN	Pauci-immune complex proliferative extracapillary and necrotizing glomerulonephritis	3.97	149	101.3	Proteinuria	Microhematuria	Initiated with IV MP at 30 mg, followed by oral MP at 32 mg/day. RTX was added via intravenous infusion at a dose of 300 mg combined with oral TGP	The patient’s kidney function has improved.
Total	30	19M/11F	60.57 ± 11.98	–	–	Confirmed	An IgG4+/IgG+ plasma cell ratio of greater than 40% and an IgG4+ plasma cell count exceeding 10 per high-power field (HPF)	–	The pathological findings were consistent with ANCA-associated glomerulonephritis.	8.11 ± 12.51	312.82 ± 130.72	63.20 ± 44.12	–	–	GC+CYC/GC+RTX/GC monotherapy/GC+CYC+RTX	/

GC, glucocorticoid; CYC, cyclophosphamide; RTX, rituximab; PS, prednisone; MP, methylprednisolone; RRT, renal replacement therapy; PLEX, plasma exchange; TGP, Tripterygium glycoside tablets; IV, intravenous; SCr, serum creatinine; IV RTX, intravenous rituximab.

Research indicates that a small number of patients have an overlap of IgG4-RD and ANCA-GN. These patients often present with severe acute kidney failure and systemic inflammatory responses. The IgG4 subclass of ANCA has been considered a potential pathogenic factor, and some previous studies on AAV-IgG4-RD overlap have reported both IgG1 and IgG4 ANCA (12, 13). However, in our patient’s case, data on the relevant serological subclasses were not available. IgG4-RD is a multi-organ disease, and renal involvement is reported in 10%–27% of cases, often accompanied by decreased complement C3 and/or C4 levels and elevated serum IgG and IgG4 levels (14, 15). Patients with IgG4-RD and low complement levels tend to have a more severe disease course and are more likely to have renal damage (16, 17). However, our patient did not show a significant drop in complement levels; instead, elevated C1q and rheumatoid factor were observed. Imaging revealed multiple pulmonary nodules; however, their stable appearance on follow-up, despite intensive immunosuppression, suggests that they may represent quiescent inflammatory sequelae rather than active extra-renal involvement of either AAV or IgG4-RD.

For the majority of patients with ANCA-GN, the standard treatment protocol involves aggressive induction therapy with GCs in combination with either RTX or CYC to quickly control the disease. This is followed by long-term maintenance therapy with less toxic drugs such as RTX or azathioprine to prevent relapse. When ANCA-GN coexists with IgG4-RKD, treatment decisions become more complex due to the distinct immunopathogenesis and standard treatment strategies of the two diseases. In clinical practice, individualized treatment approaches have been attempted, such as GC monotherapy (18), as well as the unique regimen in this case that combined GC, RTX infusions, and oral TGP. Although GC monotherapy is a first-line treatment for IgG4-RKD, its efficacy is insufficient for ANCA-GN. RTX, a B-cell depleting agent, has been mentioned multiple times in the context of patients with coexisting ANCA-GN and IgG4-RKD. It can be used in combination with GC or as part of a triple-therapy regimen with GC and CYC in severe cases (12, 19). RTX targets and depletes CD20-positive B cells, effectively preventing newly formed B cells from differentiating into IgG4-secreting plasma cells. By clearing their B-cell precursors, RTX inhibits the production of IgG4-secreting plasma cells (20). In terms of safety, RTX is superior to CYC, with lower toxicity and fewer infection-related complications. In a 4-year follow-up study, RTX-based regimens demonstrated sustained clinical remission and histological improvement (21). Given the risk of organ-threatening damage from ANCA-GN, the current treatment strategy primarily follows the guidelines for AAV, using a combination of RTX and GCs for induction of remission. The choice of RTX is particularly rational because it targets the B-cell lineage, and B-cell activation is a common core pathophysiological mechanism driving both ANCA production and the lymphoplasmacytic infiltration observed in IgG4-RD. The patient achieved satisfactory clinical and serological remission, with both the ANCA titers and serum IgG4 levels returning to normal. This provides strong evidence for the effectiveness of our treatment strategy. This case suggests that, for patients with this overlap syndrome, an aggressive treatment approach tailored for ANCA-GN may be both necessary and effective for simultaneously controlling the pathological processes of both diseases.

Recent immunological studies suggest that the co-occurrence of ANCA-GN and IgG4-TIN may not be coincidental, but rather linked to their shared immunologic mechanisms. Shiratori-Aso et al. noted that neutrophil extracellular traps (NETs) play a central role in the pathogenesis of AAV, promoting autoantigen exposure and ANCA production (22). Concurrently, Xu et al. highlighted that the immunological features of IgG4-RD are primarily mediated by Tfh2 cells and cytokines such as IL-4 and IL-21, which drive IgG4 class switching and sustain a chronic inflammatory state (23). On the other hand, Buglioni et al. observed transient MPO-ANCA positivity in only a small number of patients in an IgG4-RKD cohort, suggesting that ANCA positivity might occasionally reflect an accompanying phenomenon or an immunological “mimicry” rather than a true disease overlap (24). Collectively, the coexistence of ANCA-GN and IgG4-TIN may indirectly reflect a crossover between NET-mediated autoantigen exposure and a Tfh2-biased IgG4 response.

In our systematic review of the existing literature, we identified 30 cases of MPO-ANCA-GN that concurrently exhibited dense IgG4-positive plasma cell infiltration—a hallmark traditionally reserved for IgG4-RD. Our analysis revealed that this “IgG4-rich” phenotype is frequently associated with more severe interstitial injury. While the 2019 ACR/EULAR criteria strictly exclude ANCA-positive cases to ensure cohort homogeneity in research, the recurring clinical observation of this dual pathology suggests that the immunopathogenesis of AAV and IgG4-RD may not be mutually exclusive. Instead, they may represent a convergent spectrum of fibro-inflammatory responses (25). However, other studies have suggested that these findings may represent a diagnostic pitfall and that the presence of specific histologic features in renal masses—such as dense lymphoplasmacytic infiltrates, increased IgG4-positive plasma cells, storiform fibrosis, interstitial karyorrhexis/necrosis, neutrophilic infiltration, and glomerulonephritis—can facilitate a more accurate differential diagnosis of AAV from IgG4-TIN (26). In real-world clinical practice, however, complete distinction between AAV and IgG4-RD remains challenging when the histologic findings meet the criteria for both entities. In this case, the absence of interstitial karyorrhexis/necrosis, along with inflammation extending to the renal capsule and features of IgG4-TIN, rendered a clear distinction between AAV and IgG4-TIN challenging.

While definitive diagnosis of AAV remains challenging and the possibility of a true overlap syndrome cannot be excluded in this patient, careful clinicopathological correlation is essential in clinical practice to differentiate true overlap cases from immunological mimicry. While our case provides a valuable observation window, its single-case nature prevents us from drawing definitive conclusions about the pathogenic mechanism. This limitation underscores a clear direction for future research. At the mechanistic level, in-depth immunological analysis of renal tissue using single-cell RNA sequencing and spatial transcriptomics is a critical approach to elucidate the key cell subsets and molecular drivers behind the pathogenesis of this overlap syndrome.

## Conclusion

Currently, there is no direct evidence of a causal pathogenic link between IgG4-RD and ANCA-GN. When the histologic findings meet the diagnostic criteria for both AAV and IgG4-RD, this may reflect a shared underlying immune dysregulation. Further clarification of this relationship will require more prospective studies, mechanistic investigations, and the accumulation of data from multicenter case series.

## Data Availability

The original contributions presented in the study are included in the article/Supplementary Material. Further inquiries can be directed to the corresponding author.
